# Authors Reply to: Research Using Social Media to Recruit Research Participants Should Proceed With Caution. Comment on “Telemanagement of Home-Isolated COVID-19 Patients Using Oxygen Therapy With Noninvasive Positive Pressure Ventilation and Physical Therapy Techniques: Randomized Clinical Trial”

**DOI:** 10.2196/37413

**Published:** 2022-05-02

**Authors:** Aya Sedky Adly, Mahmoud Sedky Adly, Afnan Sedky Adly

**Affiliations:** 1 Faculty of Engineering and Technology, Badr University in Cairo (BUC) Cairo Egypt; 2 Faculty of Computers and Artificial Intelligence Helwan University Cairo Egypt; 3 Royal College of Surgeons of Edinburgh Scotland United Kingdom; 4 Faculty of Oral and Dental Medicine Cairo University Cairo Egypt; 5 Faculty of Physical Therapy, Cardiovascular-Respiratory Disorders and Geriatrics, Laser Applications in Physical Medicine Cairo University Cairo Egypt; 6 Faculty of Physical Therapy, Internal Medicine Beni-Suef University Cairo Egypt

**Keywords:** telemedicine, oxygen therapy, noninvasive positive airway pressure, BiPAP, osteopathic medicine, physical therapy, SARS-CoV-2, COVID-19, teletherapy, telemanagement

We would like to thank the authors for their interest in reading our published paper [1,2], and we would like to clarify the following points.

First, the study design and patient recruitment technique have been illustrated in sufficient detail in the methods section. Although there is a part that discusses the influence of the telemanagement system on health care development, the aim of this study was to compare two nonpharmacological respiratory treatment methods for home-isolated patients with COVID-19 by using a newly developed telemanagement health care system. The influence of the telemanagement system on health care development has been further discussed in many other articles [3-6], and its discussion was therefore considered outside of the scope of our paper.

Second, concerning the health conditions of the study patients, we disagree with the comment because these variables were considered in both this study and its analysis. As noted in the inclusion criteria, only the American Society of Anesthesiologists Class-I patients were deemed eligible for participation in the study before onset of COVID-19. We have also stated in the results section that the patients’ age ranged between 21 and 40 years.

Third, the eligibility of the recruited patients, inclusion criteria, and exclusion criteria have already been well addressed in the methods section by specifically defining the selection criteria to avoid any unwanted effect of confounding factors.

Fourth, both the sample size and power calculations have already been presented in the statistical analysis section.

Fifth, we also disagree with the comment concerning the snowball subject recruitment technique, since it is considered an efficient sampling technique that is used to recruit participants who are hard to be located.

Sixth, we have reviewed the mentioned reference about the echo chamber effect on social media and found that it discusses public opinion formation and not patient recruitment. Thus, for this suggestion, we would expect to be provided with evidence on the suitability of a comprehensive database for patient recruitment. We would also want to clarify that all the selected patients met the study’s eligibility criteria, and in the methods section, we have explained how we ensured this.

Seventh, studying the influence of the implementation of telemanagement together with the national health care database on health care development was not the aim of this study.

Eighth, we believe that the research design was thoroughly explained with sufficient detail that can be easily understood by any health care worker. However, for a suggestion on design enhancement, we would expect to be more specific on what points the design should be enhanced, along with an acceptable reason.

Moreover, for the suggestion to implement patient recruitment using more suitable techniques, we would expect to be provided with evidence that the patient recruitment technique used in our study is inferior to other techniques or that it has a negative effect on the study outcomes, which is not the case.

In addition, the influence of the telemanagement system on health care development has been addressed by many studies and is beyond the scope of this publication.

Finally, we appreciate this chance to reply to this letter and would like to advise researchers to consider recruiting larger study samples before adopting our techniques.
